# Aroma Release and Consumer Perception During Cider Consumption

**DOI:** 10.3390/foods14061005

**Published:** 2025-03-16

**Authors:** Yuqing Chen, Ruoqing Zhao, Guoxin Jiang, Guanchen Liu, Yanyun Cao, Xiao Ye, Yuezhong Mao, Li He, Yong Cheng, Shiyi Tian, Zihan Qin

**Affiliations:** 1 School of Food Science and Biotechnology, Zhejiang Gongshang University, Hangzhou 310018, China; 2 Zhejiang-UK Joint Research Laboratory of Food Sensory Science, School of Food Science and Biotechnology, Zhejiang Gongshang University, Hangzhou 310018, China; 3 Innovation Center of Yangtze River Delta, Zhejiang University, Jiaxing 314100, China; 4 Hangzhou Skyherb Technologies Co., Ltd., Hangzhou 313399, China

**Keywords:** apple cider, retronasal olfaction, sensory analysis, consumers preference, aroma release

## Abstract

Flavor is an important property affecting consumer acceptance, yet little is known about the correlation between the sensory attributes, volatile compounds, and consumer preference during cider consumption. This study was the first to evaluate which sensory attributes of commercial apple ciders in China were preferred by Chinese consumers. Meanwhile, GC-MS and GC-IMS were conducted to characterize the aroma release both in orthonasal and retronasal olfactory perceptions. The sensory analyses exhibited that Chinese consumers preferred “sweet cider”, and sensory attributes such as “a-tropical fruit”, “f-fruity” and “t-sweet” were the most popular. A total of 63 volatile compounds were identified using GC-MS, while both the variety and concentrations of these compounds detected by GC-IMS were lower. Finally, partial least squares (PLS) analysis was used to establish two models based on sensory data, and orthonasal and retronasal volatile compounds. The two models had 32 and 29 compounds with variable importance in projection (VIP) values > 1, respectively. The results revealed that the compounds with high correlation with “t-sweet” and “f-fruity” were roughly the same in two PLS models, whereas the number of compounds contributing positively to “t-sour” and “f-fermented” changed significantly.

## 1. Introduction

Cider, a fruit wine made by fermenting apple juice with an alcohol content ranging from 1.2% to 8.5% (*v*/*v*), has become the world’s second most consumed fruit wine [1]. Originally processed in several countries in Europe, mainly in the United Kingdom, France, and northern European countries, it is now also produced in Argentina, Australia, South Africa, and China. The flavor is the most critical quality attribute of apple ciders and serves as the primary indicator for evaluating its overall sensory quality, ultimately influencing the preferences of potential consumers [2]. The volatile profiles of apple ciders have been extensively studied over the past 50 years, and more than 300 volatile compounds belonging to 10 chemical classes have been identified [3]. The results have presented alcohols, esters, aldehydes, ketones, and acids as the most abundant volatile classes in apple ciders, and are typically characterized by their fruity, fermentative, cooked apple, and alcoholic aromas. As China is the world’s largest producer of apples, cider is gaining importance in the Chinese market [4]. However, there is no available data concerning the unique flavor characteristics of the commercial apple ciders in the Chinese market.

After volatile compounds are released from food or beverages, they can be perceived via two pathways: the orthonasal and the retronasal routes. Anterior nasal olfactory perception refers to odors from the external environment entering the olfactory receptor cells via the front of the nasal cavity. The retronasal route involves aromatic compounds within the oral cavity ascending through the oropharyngeal pathway to the olfactory mucosa during eating, drinking, swallowing, and exhalation [5]. Numerous studies demonstrate significant differences in both the type and quantity of aroma compounds released via the orthonasal compared to the retronasal route [6,7,8]. Olfactory stimulation resulting from the two modes of olfaction is affected by different external influences and physiological factors, leading to diverse perceptions between them [7]. In the research conducted by Shuo et al. (2020), they found aroma compounds with milk fat exhibit higher intensity when perceived retronasally, whereas the acidic aromas demonstrate greater intensity during orthonasal perception [6]. Genovese et al. confirmed that both orthonasal and retronasal perceptions play an important role in the evaluation of the quality of wine [8]. The changes that odorant molecules might experience in the oral cavity during wine consumption are an incipient area of research that is providing new and valuable findings.

The extremely brief oral processing time of liquid foods presents a significant challenge for the collection of retronasal aroma. Several techniques have been described in the literature for studying the in vivo release of aroma compounds during food consumption. On-line in vivo analytical approaches, such as atmospheric pressure chemical ionization mass spectrometry (API-MS) and proton transfer reaction mass spectrometry (PTR-MS), are often used, reflecting the patterns of aroma release in real time during wine consumption. However, they are difficult to apply to real food and beverage matrices, such as wines characterized by complex flavor profiles, since they cannot provide clear compound identification [9]. Moreover, off-line in vivo analyses, including retronasal aroma trapping device (RATD), intra-oral solid-phase microextraction (SPME) technique, and gas sampling bags coupled with gas chromatography–mass spectrometry (GC-MS), have also been developed [10,11]. Nonetheless, the high time cost of these approaches and the requirement of additional equipment make their implementation in many labs very difficult. At present, solid-phase microextraction–gas chromatography–mass spectrometry (SPME-GC-MS) has been widely used to analyze aroma compounds in many types of fruit wine. Combined with the odor activity value (OAV), it is possible to study the potential key aroma compounds and their contributions to the flavor patterns of cider [12]. Gas chromatography ion mobility spectrometry (GC-IMS) is an emerging analytical technology with high sensitivity and high separation efficiency for detecting volatile organic compounds (VOCs), which fully combines the high-resolution capability of GC technology with the high discrimination capability of IMS technology [13]. Compared to GC–MS, GC-IMS provides faster and more convenient aroma analysis, allowing for investigation of aroma release and retronasal perception [14]. The integration of SPME-GC-MS and GC-IMS provides a more comprehensive and accurate approach to aroma analysis under various conditions. GC–MS allows for the identification of a broader range of compounds, while GC-IMS, characterized by its high sensitivity and rapid response, provides a more in-depth analysis of the volatile components. This study represents the first application of a combined approach involving GC-IMS and GC–MS to systematically compare the VOCs of apple ciders to explore the aroma release and retronasal olfactory perception during cider consumption.

The food industry regards sensory analysis and consumer behavior research as the most valuable tools during the new product development process, ensuring successful product innovation and consumer satisfaction [15]. However, although apple cider products are sold in some areas of China, it is not known what aromas in ciders are preferred by Chinese consumers. Accordingly, this study evaluated which sensory attributes of five apple ciders in China were preferred by a panel of Chinese consumers. GC-MS and GC-IMS were conducted to characterize the aroma release both in orthonasal and retronasal olfactory perceptions during cider consumption. PLSR correlation analysis was used to explore the sensory attributes and key aroma compounds variations among different cider products, as well as their correlation.

## 2. Materials and Methods

### 2.1. Cider Samples

Five typically commercial apple ciders were selected and purchased via online shopping. These samples covered a wide range of sensory characteristics, alcohol content, and production methods. They represented the ciders accessible to Chinese consumers. Table 1 shows the cider samples and their basic information.

### 2.2. Chemicals

4-Methyl-1-pentanol (≥97.0%) and alkanes standard solution C8–C20 (99.9%) were purchased from the Sigma-Aldrich Chemical Co., Ltd. (Shanghai, China).

### 2.3. Quantitative Descriptive Analysis

Quantitative descriptive analysis (QDA) was conducted at the Collage of Food and Bioengineering at Zhejiang Gongshang University. A group of ten panelists (four male and six female, 22–28 years old) were selected from thirty candidates. All panelists had at least one year of prior experience in the sensory evaluation of foods and had demonstrated satisfactory performance. The participants gave their written consent to join the sensory test, after receiving the full information. During sessions 1 and 2, the panelists generated sensory terms based on the perceptions of cider samples, using a list of attributes from previous studies as additional guidance. A total of 17 attributes were selected by consensus to describe the cider samples, including 8 for the aroma, 3 for flavor, 3 for taste, 2 for mouthfeel, and 1 for aftertaste properties. Table 2 presents a complete list of aroma, flavor, taste, mouthfeel, and aftertaste terms and their reference standards. In session 3, panelists were presented with 15 cm linear scales and appropriate anchors were selected. A practice session was held in the fourth training session using the same tasting procedure as that used in the formal evaluation, which was used to check the panelists’ performance and help them to be familiar with the setup of the formal evaluation sessions. During the sensory evaluation, 20 mL of each apple cider sample were each put in a covered and odorless 50 mL glass cup, and the glasses were submitted to the panelists with random three-digit codes. The intensities of the 17 sensory attributes were scored using 15 cm linear scales, anchored with “Not Perceived” at the left end and “Very Intense” at the right end. The analyses were conducted in triplicate.

### 2.4. Check-All-That-Apply

#### 2.4.1. Sensory Lexicon

To gather information on consumers’ sensory perception of apple ciders, a multiple-choice questionnaire containing 21 attributes was provided to consumers, which were “fresh apple”, “cooked apple”, “tropical fruit”, “pear”, “citrus”, “fermented”, “malty”, “floral”, “honey”, “jujube”, “rusty”, “moldy”, “chemical”, “dusty”, “alcohol”, “sweet”, “sour”, “bitter”, “astringent”, “tingling”, and “bubbly”. The descriptors were based on a list generated by the trained panel and previous studies on consumer preference analysis for cider and wine [16,17].

#### 2.4.2. Procedure

A total of 60 consumers participated in the CATA test. Samples were coded with three random digits and presented in a random order. After each evaluation, participants were instructed to rinse their mouths with water after each tasting and eat unsalted crackers between samples to try to make the palate conditions similar for each sample.

Participants were asked to first smell and then taste ciders. They began by evaluating a hedonic question, using a 9-point hedonic scale ranging from 1 (“dislike extremely”) to 9 (“like extremely”) to rate their overall liking. Then, they answered the CATA questions. Finally, after completing the cider evaluation, consumers were asked to complete demographic questions, such as gender, age, and drinking frequency.

### 2.5. Aroma Analysis

#### 2.5.1. Volatile Compounds of Cider

The volatile compounds were measured using HS-SPME-GC-MS [12]. A 5.0 mL sample of apple cider was added to a 20 mL headspace injection vial (Supelco Inc., Bellefonte, PA, USA). At the same time, 40 µL of 4-methyl-1-pentanol (2 mg/mL) was added as an internal standard. Subsequently, the sealed injection vial was placed in a 60 °C water bath for 12 min stabilization and the SPME fiber (divinylbenzene/carboxen/polydimethylsiloxane, Supelco, Inc., Bellefonte, PA, USA) was inserted into the vial and exposed to 60 °C headspace for 40 min. After adsorption, fiber was inserted into an Agilent 8890-5977C gas chromatograph and mass spectrometer (Agilent Technologies, Inc., Palo Alto, CA, USA) and desorbed at 240 °C for 10 min. Volatile compounds were separated and identified using a capillary column (HP-5MS, 30 m × 0.25 mm i.d, 0.25 µm film thickness).

The oven temperature was kept at 40 °C for 1 min, then raised to 160 °C at a rate of 3 °C/min, then raised to 230 °C at a rate of 8 °C/min, and then held at 230 °C for 10 min.

The component assignment was performed through computer matching with data in the NIST14 library. For the semi-quantitation of the analytes, relative content of individual component was calculated using the concentration of the internal standard multiplied by the ratio of the peak area of the volatile compound to the peak area of the internal standard.

#### 2.5.2. Oral Aroma Detection After Drinking Cider

The FlavorSpec GC-IMS system (G.A.S., Dortmund, Germany) was used to analyze the composition of volatile compounds in the gas after drinking apple cider. Specifically, 10 mL cider was put into the mouth, and the panelists were asked to swallow it after 10 s, during which they held their breath, then blew oral air into the air bag, and then squeezed the gas into the inlet for detection. The injection volume was 1 mL, and 3 parallel groups were measured for each sample. During the interval, panelists had 5 min to rinse their mouths, drink water, and take a rest.

The carrier gas flow rate program was referenced from previous work [18]. The initial flow rate was 5 mL/min, holding for 2 min, then increased to 45 mL/min within 8 min, then increased to 100 mL/min within 2 min, and then increased to 150 mL/min within 8 min. The ionization source of the IMS was 3H (300 MBq). The temperature of the drift tube in the IMS was 45 °C and the flow rate of carrier gas was 150 mL/min.

### 2.6. Statistical Analysis

All experiments adopted a parallel experiment of three repeats. Data were analyzed by one-way analysis of variance (ANOVA) with SPSS 19.0 (SPSS Inc., Chicago, IL, USA), as well as Duncan’s post hoc test to test whether the differences between samples were statistically significant. Origin 2025 (OriginLab Corporation, Northampton, MA, USA) was used for the data plots.

The correspondence analysis (CA) of the CATA questionnaire results and the partial least squares (PLS) analysis were performed using XLSTAT 2023.1.4 software (Lumivero, LLC, Denver, CO, USA). GC-IMS data were obtained and analyzed by VOCal software (v0.4.03) from G.A.S.

## 3. Result and Discussion

### 3.1. Sensory Evaluation

**Figure 1:**
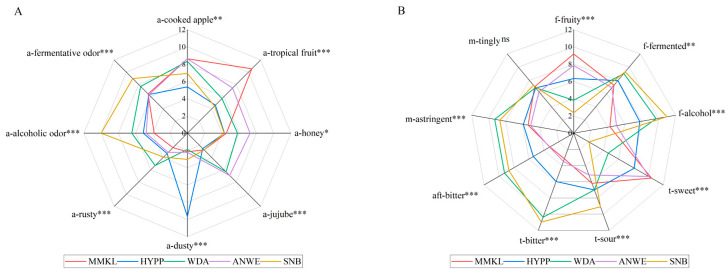
Sensory profile of 5 apple ciders. (**A**) Sensory profile of aroma. (**B**) Sensory profile of flavor, taste, mouthfeel, and aftertaste. “ns” indicates no significant difference (*p* > 0.05); *, **, and *** represent a significance at *p* < 0.05, *p* < 0.01, and *p* < 0.001, respectively.

To determine the sensory difference among the cider samples, quantitative descriptive analysis was performed, and Figure 1 shows the statistical results. It can be seen that there were distinct differences (*p* < 0.05) in the intensity of all the aroma, flavor, taste and mouthfeel attributes, except for the M-Tingly. Notably, the distinctions among the five cider samples were quite pronounced. The MMKL sample exhibited a more distinct tropical fruit aroma, fruity flavor, and sweet taste. HYPP was characterized by a stronger dusty aroma, while the WDA sample was notably rusty, fermented, and astringent. ANWE stood out with its honey note, and SNB was particularly marked by alcoholic and fermentative aromas, as well as its alcoholic flavor, and sour and bitter taste. Among them, the scores of tropical fruit, alcoholic, sweet, and bitter were the highest, which is consistent with the previous study [19].

**Figure 2:**
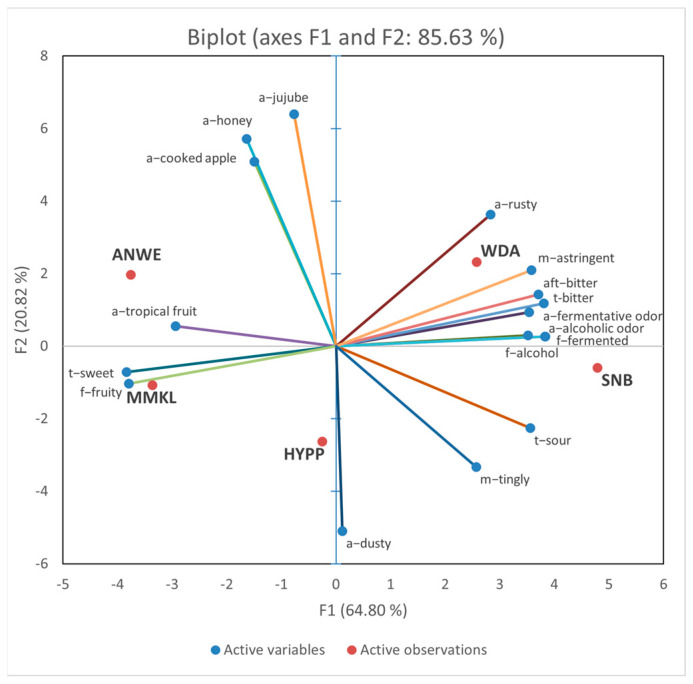
Principal component analysis (PCA) bi-plot for sensory attributes and 5 apple ciders.

Figure 2 shows the PCA images of the sensory characteristics of these cider samples. The first principal component (PC1) accounted for 64.80% of the variance, and PC2 accounted for 20.82%. The PCA bi-plot explains 85.63% of the total variations in the first two dimensions. The WDA and SNB samples were both distributed on the right side along the PC1 axis, positively correlated with the “f-alcohol”, “t-bitter”, “aft-bitter”, “a-fermentative odor”, and “m-astringent” descriptors. The ANWE and NMKL samples were distributed on the left side and positively correlated with the “t-sweet”, “f-fruity”, and “a-tropical fruit” descriptors. The HYPP samples were distributed in the negative side of PC2, with obvious “a-dusty”, “m-tingly”, and “t-sour”.

### 3.2. Consumer Perception

One of the main interests in the brewing industry is to understand the flavor characteristics most desired by consumers in cider. This experiment was conducted through a group of Chinese consumers, recording their overall liking and flavor perception of five apple ciders.

**Figure 3:**
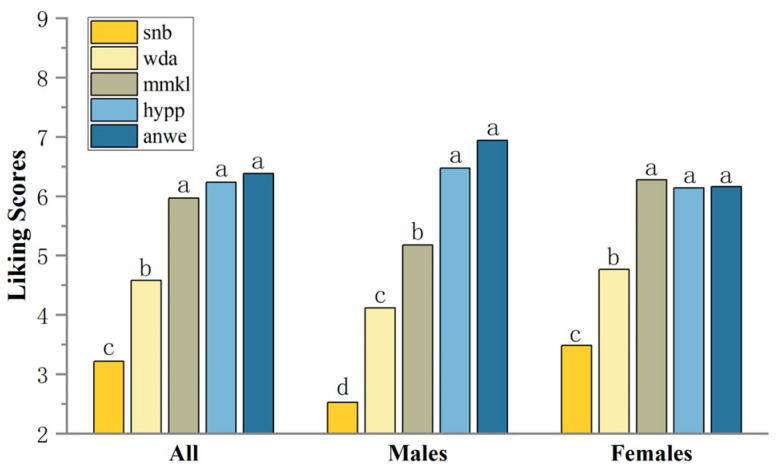
Mean liking scores of consumers. Different letters indicate significant differences at *p* < 0.05.

Consumer overall liking scores ranged from 3.22 to 6.38, with ANWE being the highest rated apple cider (Figure 3). Considering the differences in consumer ratings, consumers were categorized by gender based on demographic information, resulting in a male cluster of 17 and a female cluster of 43. Interestingly, the preference rankings for apple ciders differed between males and females. The MMKL sample, which received the highest score in the female group, was ranked in the statistically significant second tier (*p* < 0.001) among the male group. Moreover, males showed a stronger preference for the ANWE cider sample. For the lowest ranked SNB sample, the scores were significantly lower in the male group.

**Figure 4:**
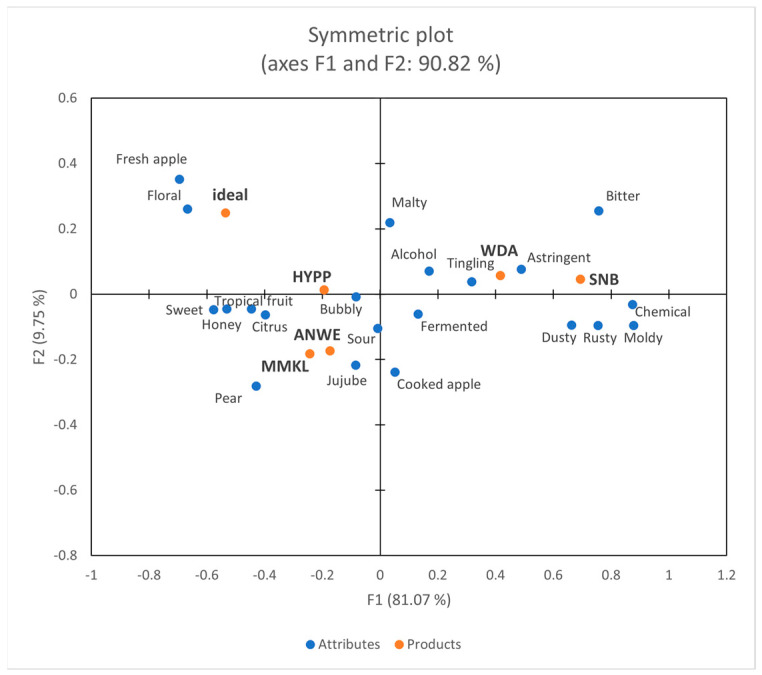
Correspondence analysis of Check-All-That-Apply (CATA) data.

Figure 4 depicts a projective map for the correspondence analysis (CA). The first two dimensions collectively explained 90.82% of the variability in the data. Dimension 1 accounted for 81.07% of this variability, whereas dimension 2 accounted for 9.75%. Dimension F1 distinguished between HYPP, WDA, and SNB, whereas dimension F2 separated ANWE and MMKL from other ciders. The results of CATA aligned with the sensory data obtained by the trained panel. In addition, “Ideal” represented the attributes of the consumers’ ideal product, which were desired to have “fresh apple”, “floral”, and “sweet”. This finding was consistent with a recent study on wine among Chinese consumers [20].

Through consumer analysis, it was revealed that males tend to favor products with “bubbly” and “sour”, while females prefer products with “sweet” and “tropical fruit”. They consistently dislike products with “bitter”, “astringent”, and “chemical”, which was consistent with previous research on Chinese consumers’ preference for fruit wines [21].

### 3.3. Aroma Release During Cider Consumption

#### 3.3.1. Volatile Compounds of Cider

In our study, cider sample consisted of 63 volatile compounds. Table 3 summarizes the result of volatile compounds in the five commercial ciders, including 30 esters, 12 alcohols, 6 acids, 5 aldehydes, and 4 ketones.

Esters play a crucial role in cider flavor and represent the largest category of volatile compounds. Among them, ethyl octanoate was the most abundant ester in cider, with a concentration as high as 20.554 mg/L. Additionally, ethyl hexanoate, hexyl acetate, phenylethyl acetate, and ethyl decanoate also had high concentrations, ranging from 1.722 to 10.067 mg/L, 0.352 to 8.288 mg/L, 0.202 to 17.071 mg/L, and 0.733 to 9.900 mg/L, respectively.

Ethyl esters are a notable group of volatiles that contribute to the desirable fruity flavor of cider. HYPP exhibited the highest concentrations of ethyl octanoate, ethyl hexanoate, and ethyl decanoate, which may impart fruity, green apple, brandy, and wine-like aromas to this cider [22]. Ethyl laurate is regarded as a key compound for identifying sparkling wine types, and its concentration in HYPP exceeded the odor threshold [23]. Ethyl butyrate, presented in all five samples, showed the highest concentration in MMKL. This compound provides apple and pineapple aromas, which could explain the tropical fruit flavor characteristic of MMKL. SNB showed the highest concentration of ethyl lactate, which contributes fruity and milky notes to cider. However, its odor detection threshold is relatively high (approximately 14 mg/L) [24], making it less impactful on the wine aroma compared to ethyl acetate. Acetate esters are also important aroma components in apple cider. Ethyl 2-methylbutanoate and isoamyl acetate, which have low odor thresholds, are the main acetate esters in cider [25]. These compounds were detected at their highest concentrations in MMKL, imparting a banana-like fruity aroma that is believed to enhance the fruity and sweet flavor of cider [23,26]. Moreover, MMKL exhibited the highest concentration of phenylethyl acetate among all samples, a compound known for its floral and pleasant fragrance [27]. This aligned with previous sensory evaluation results, where MMKL scored the highest in “a-tropical fruit”.

Alcohols are among the most abundant volatile compounds found in all cider samples. Higher alcohols are regarded as one of the most important precursors of esters, but they may have adverse effects on the quality of cider and wine at excessive levels [2,28]. In this study, the concentration of higher alcohols was within the acceptable range. Phenylethyl alcohol was found at significantly high levels across different ciders, reaching as high as 95.982 mg/L in the SNB sample, possibly related to the higher alcohol content of this sample. Phenylethyl alcohol has rose and honey-like aromas and can contribute positively to the aroma of cider [29]. The second most abundant alcohol is hexanol, which has also been observed to have a high concentration in published studies on apple cider [30], indicating that hexanol plays an important role in the aromatic profile of apple cider. Additionally, 2,3-butanediol, which has fruit and butter aromas [1], was detected in the ANWE, WDA, and SNB samples.

Acids were found in lower concentrations in the samples and only showed high levels in certain samples. The short-chain fatty acid hexanoic acid was present in ANWE, WDA, and SNB. Medium-chain fatty acids were found at higher levels in SNB, with octanoic acid being the primary contributor, while ANWE cider contained a high concentration of decanoic acid. Overall, acids were abundant in ANWE, WDA, and SNB, which may contribute to the perception of “a-cooked apple” and “a-fermentative odor” in cider. However, it is worth noting that the short-chain fatty acids of C3–C5 can emit unpleasant odors, often associated with cheese or rancid aromas [22]. Volatile acids are important for aromatic complexity in apple ciders and they can contribute to the vinegar-like, sweat, and rancid notes of apple ciders [19].

Among the minor volatile compounds, the main detected aldehydes included 3-furfural, benzaldehyde, nonanal, and decanal. *β*-Damascenone, which imparts roasted apple, floral, and honey-like aromas [25,31], was found at the highest concentration in WDA, giving this sample a more pronounced honey aroma. Among the phenolic compounds, eugenol was identified as one of the most important. Additionally, studies have shown that even when the concentration of phenolic compounds exceeds their thresholds, volatile phenols and similar compounds do not independently influence the flavor of wine [32].

#### 3.3.2. Oral Aroma Release

**Figure 5:**
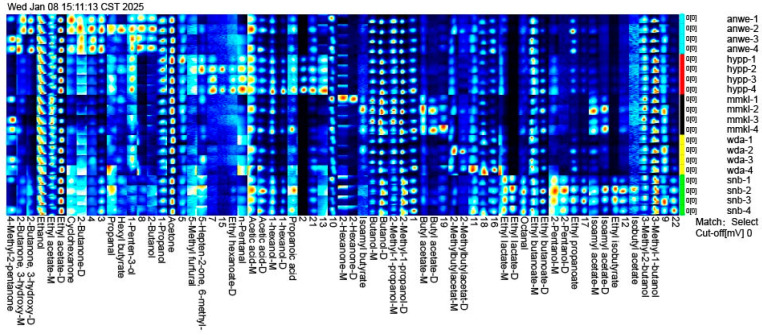
The gallery plot of oral volatile compounds identified by GC/IMS.

Figure 5 shows two-dimensional chromatogram results of aroma release in the oral aroma release. A total of 62 volatile compounds were detected in ciders, including 9 alcohols, 14 esters, 9 ketones, 4 aldehydes, 2 acids, 1 furan, and 24 unidentified compounds. The concentration and diversity of retronasal compounds were lower than the aroma detected by GC-MS, as the aroma compounds were absorbed by saliva proteins and the oral mucosa during consumption [33].

Several aroma compounds, such as 3-methyl-1-butanol, acetic acid, 2-methyl-1-propanol, and butanol, were present in all cider samples at varying concentrations, likely because these substances are fundamental volatiles produced during the cider fermentation process [34]. Ethyl acetate was found to exhibit the highest concentration in oral aroma and accounted for the largest proportion of post-drinking aroma compounds. In addition, isobutyl acetate, ethyl isobutyrate, and 2-pentanol, were detected only in the oral aroma of SNB. Isoamyl acetate and butyl acetate were detected exclusively in MMKL. Ethyl hexanoate and n-pentanal were found only in HYPP, with concentrations significantly higher than the other samples. These compounds contributed unique aromas to the cider consumption in mouth.

Interestingly, compared to the volatile compounds identified by GC-MS, isoamyl acetate and ethyl hexanoate were detected at concentrations exceeding their thresholds in all samples. However, in oral aroma compounds, isoamyl acetate was detected only in MMKL, while ethyl hexanoate was detected only in HYPP. This aligns with the findings from other studies, which reported that ethyl hexanoate has weak binding with the oral mucosa [10]. Additionally, although 2-methylbutylacetat was detected at the highest concentration in MMKL, it was found at the highest level in the post-drinking oral gas of WDA. These compounds are believed to contribute more fruity and sweet flavors to cider [24].

In conclusion, the retention of volatile compounds in the oral gas after drinking may not only be influenced by their concentration but is also closely related to the chemical properties of the aroma compounds [8].

### 3.4. Correlations Between Sensory Perception and Aroma Release During Cider Consumption

**Figure 6:**
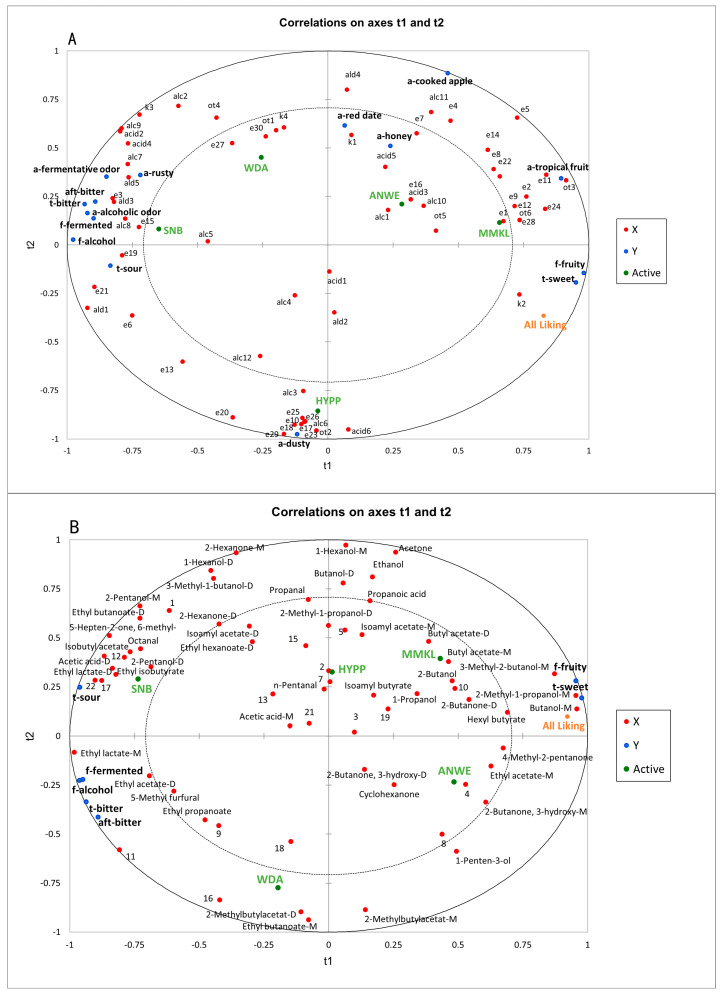
(**A**) Correlations between sensory attributes and volatile components. (**B**) Correlations between the sensory perception and the oral volatile compounds.

As Figure 6A shows, partial least squares (PLS) analysis was conducted to establish the relationships between the sensory attributes and the volatile compounds of the five ciders based on SPME-GC-MS analysis. The PLS analysis was performed using 63 volatile components and 15 sensory descriptors, excluding mouthfeel attributes. Two principal components (t1 and t2) explained 80.86% of the data variation, indicating that these components could effectively explain the dataset. X represented volatile compounds, Y represented sensory attributes, and active denoted the different cider samples. In model A, there were 32 compounds with variable importance in projection (VIP) values > 1, including ethyl lactate, diethyl succinate, ethyl phenylacetate, ethyl hexanoate, lactic acid isoamyl ester, octanoic acid, and hexanoic acid. These illustrate that these important volatile compounds played a crucial role in distinguishing different apple ciders [35].

As Figure 6A shows, there was a very clear differentiation among the five apple ciders. MMKL and ANWE were located on the positive t1 and positive t2. These two ciders could be called “sweet ciders”, which were rated high in “a-tropical fruit”, “a-cooked apple”, “f-fruity”, “t-sweet” attributes, and overall liking score, indicating a positive correlation between fruity aroma and consumer preference, which was consistent with the sensory evaluation results. Moreover, these aromas were mainly described by positive contributions from ethyl butanoate, butyl acetate, isoamyl acetate, hexyl acetate, 2-methylbutyl acetate, and phenethyl acetate. The relationships observed between fruity descriptors and acetate esters were also found in another study on the prediction of sensory properties of apple [36]. HYPP was distinct from other ciders, characterized by a unique aroma profile. The reason for the lower overall liking score of this sample may be associated with an unpleasant “a-dusty” presented by benzyl alcohol, dodecanoic acid, ethyl laurate, and ethyl decanoate. SNB displayed a stronger “f-fermented”, “f-alcohol”, and the lowest preference score. In addition to diethyl succinate, ethyl phenylacetate also had a high correlation with “t-sour”. This is consistent with the conclusions of sensory characteristics studies of commercial Australian wines [37]. “F-alcohol” was highly correlated with alcohols, such as phenylethyl alcohol and 1-octanol.

Figure 6B illustrates the relationships between the sensory data and the aroma compounds in the post-drinking oral aroma of ciders based on GC-IMS analysis. Here, the sensory data included only the flavor and taste attributes. Two principal components (t1 and t2) explained 96.38% of the data variation, demonstrating a high level of generalization in the model. X represented volatile compounds, Y represented sensory attributes, and active denoted the different cider samples. In this model, there were 29 compounds with VIP values > 1, including ethyl acetate, ethyl lactate, isobutyl acetate, butanol, 2-methyl-1-butanol, 2-pentanol, and acetic acid.

Model B revealed that aroma compounds in the mouth can significantly distinguish the samples. For instance, “f-fruity” and “t-sweet” were closely associated with the aroma compounds in the upper right corner, such as butyl acetate, ethyl acetate, and hexyl butyrate. This result was also demonstrated in Model A. Additionally, 3-methyl-1-butanol, 2-methyl-1-propanol, and butanol also exhibited positive contributions to “t-sweet” and “f-fruity”. In orthonasal olfactory perception, these compounds have proved to help increase fruity characteristics [34,38], and thus may be a main factor determining high consumer preference in retronasal olfactory perception.

For the taste descriptors, the most prominent “t-sour” was highly correlated with volatile compounds such as ethyl isobutyrate and isobutyl acetate, which were closely associated with citrus flavors [19]. Additionally, acetic acid and octanal also contributed positively to “t-sour”. However, certain esters and alcohols, such as phenylethyl alcohol, which exhibited a strong correlation with “a-alcohol” in Model A, were not detectable in the oral aromas. This finding aligns with previous reports on post-drinking aroma of wine [8].

## 4. Conclusions

This study firstly investigated the sensory preferences of Chinese consumers for five apple cider samples, focusing on the sensory attributes that contributed to their preferences. GC-MS and GC-IMS analyses were employed to characterize the aroma release during cider consumption, specifically through orthonasal and retronasal olfactory perceptions. The sensory analyses demonstrated that “sweet cider” was the most favored by Chinese consumers, with attributes such as “a-tropical fruit”, “f-fruity”, and “t-sweet” being particularly popular. A total of 63 volatile components were detected by GC-MS, with esters, alcohol, aldehydes, acids, and ketones being the main aromatic substances in the apple ciders. Furthermore, comparative analysis results showed that the amount and species of the aroma compounds in mouth detected by GC–IMS were less than that of GC–MS. Finally, partial least squares (PLS) analysis was used to establish two models based on sensory data, and orthonasal and retronasal aroma compounds. In model A, there were 32 compounds with VIP values > 1, including ethyl lactate, diethyl succinate, ethyl phenylacetate, ethyl hexanoate, isoamyl lactate, octanoic acid, and hexanoic acid. In model B, there were 29 compounds with VIP values > 1, including ethyl acetate, ethyl lactate, isobutyl acetate, butanol, 2-methyl-1-butanol, 2-pentanol, and acetic acid. The results revealed that the compounds with a high correlation with “t-sweet” and “f-fruity” were roughly the same in the orthonasal and retronasal aroma, such as butyl acetate, ethyl acetate, and hexyl butyrate. However, the number of compounds that contributed to the attributes of ‘t-sour’ and ‘f-fermented’ significantly increased in oral aroma release. Notably, ethyl isobutyrate, isobutyl acetate, acetic acid, and octanal displayed high correlations with these sensory attributes.

Overall, this study offers comprehensive insights into the key aroma of cider in orthonasal and retronasal olfactory perceptions, providing further support for the studying of aroma release during consumption and facilitating the control and improvement of sensory product quality.

## Figures and Tables

**Table 1:** Description of the ciders used in the study.

Sample Name	Alcoholic Content (%)	Type	Ingredients	Country of Origin
WDA	5	Dry	99.96% apple juice, SO_2_, and sulfites.	France
ANWE	2	Sweet	Apple juice, and SO_2._	France
HYPP	4.5	Semi-sweet	Apple juice, citric acid, yeast, and sodium metabisulfite.	Poland
SNB	5.5	Dry	Apple juice, CO_2_, and sulfites.	Estonia
MMKL	1.6	Semi-sweet	Apple juice, CO_2_, and sulfites.	Estonia

**Table 2:** List of sensory attributes and corresponding reference standards.

Attributes	Reference Standards
Aroma	
Cooked apple	Pieces of chopped ripe apple in boiling water for 5 min.
Tropical fruit	Banana and pineapple slices.
Honey	10 g honey.
Jujube	Dried jujube slices.
Dusty	1 g of baking powder in 10 mL of water.
Rusty	Metal spoon.
Alcoholic odor	10% alcohol.
Fermentative odor	Apple cider vinegar and white vinegar (1:1).
Flavor	
Fruity	Apple juice.
Fermented	Apple cider vinegar and white vinegar (1:1).
Alcoholic	10% alcohol.
Taste	
Sweet	20 g sucrose in 1 L water = 8, 10 g sucrose in 1 L water = 3.
Sour	0.8 g citric acid in 1 L water = 9, 0.5 g citric acid in 1 L water = 5.
Bitter	0.006 g quinine in 1 L water = 4, 0.01 g quinine in 1 L water = 12.
Mouthfeel	
Astringent	0.6 g/L aluminum sulfate.
Tingly	Sparkling water.
Aftertaste	
Bitter	Steep 4 g of lotus seed core in 500 mL of boiling water for 2 min and remove = 8.

**Table 3:** Volatile compounds identified and quantified (mg/L) by GC-MS in five apple ciders.

Code	Compounds	CAS	SNB	WDA	MMKL	HYPP	ANWE
Esters							
e1	Ethyl butanoate	105-54-4	1.14 ± 0.014 b	0.91 ± 0.050 b	3.02 ± 0.25 a	0.95 ± 0.14 b	0.90 ± 0.090 b
e2	Butyl acetate	123-86-4	nd	0.31 ± 0.026 b	1.84 ± 0.19 a	nd	0.19 ± 0.018 b
e3	Ethyl lactate	97-64-3	3.69 ± 0.091 a	0.98 ± 0.046 b	nd	0.12 ± 0.034 d	0.44 ± 0.074 c
e4	Ethyl crotonate	623-70-1	nd	0.24 ± 0.030 b	0.16 ± 0.010 c	nd	0.38 ± 0.047 a
e5	Ethyl 2-methylbutanoate	7452-79-1	0.28 ± 0.023 c	1.14 ± 0.048 b	1.40 ± 0.13 a	0.29 ± 0.036 c	1.21 ± 0.133 b
e6	Ethyl isovalerate	108-64-5	0.25 ± 0.016 a	nd	nd	0.12 ± 0.017 b	nd
e7	Isoamyl acetate	123-92-2	1.23 ± 0.082 c	3.02 ± 0.14 b	3.73 ± 0.50 a	0.29 ± 0.028 d	0.57 ± 0.076 d
e8	2-Methylbutylacetat	624-41-9	0.195 ± 0.035 c	0.46 ± 0.044 b	1.30 ± 0.12 a	nd	0.16 ± 0.039 c
e9	Pentyl acetate	628-63-7	nd	0.044 ± 0.002 b	0.33 ± 0.039 a	nd	nd
e10	Methyl hexoate	106-70-7	nd	0.024 ± 0.003 b	nd	0.17 ± 0.020 a	nd
e11	Ethyl 3-hydroxybutyrate	5405-41-4	nd	0.045 ± 0.003 b	0.177 ± 0.015 a	nd	0.058 ± 0.006 b
e12	n-Butyl butanoate	109-21-7	nd	nd	0.29 ± 0.13	nd	nd
e13	Ethyl hexanoate	123-66-0	5.25 ± 0.12 c	7.33 ± 0.38 b	1.72 ± 0.13 d	10.07 ± 1.26 a	2.13 ± 0.28 d
e14	Hexyl acetate	142-92-7	0.35 ± 0.013 d	5.50 ± 0.24 b	8.29 ± 1.32 a	0.44 ± 0.063 d	1.718 ± 0.25 c
e15	Isoamyl lactate	19329-89-6	1.04 ± 0.076	nd	nd	nd	nd
e16	Ethyl sorbate	2396-84-1	nd	nd	nd	nd	6.04 ± 0.87
e17	Methyl octylate	111-11-5	nd	nd	nd	0.80 ± 0.12	nd
e18	Ethyl benzoate	93-89-0	0.96 ± 0.074 bc	0.51 ± 0.034 cd	0.06 ± 0.008 d	4.88 ± 0.94 a	1.46 ± 0.19 b
e19	Diethyl succinate	123-25-1	11.37 ± 0.78 a	1.02 ± 0.15 c	0.28 ± 0.043 c	2.34 ± 0.27 b	0.37 ± 0.044 c
e20	Ethyl octanoate	106-32-1	7.66 ± 0.64 b	5.99 ± 0.73 bc	1.43 ± 0.068 d	20.55 ± 3.56 a	2.78 ± 0.27 cd
e21	Ethyl phenylacetate	101-97-3	0.78 ± 0.058 a	0.34 ± 0.004 c	0.071 ± 0.002 d	0.48 ± 0.082 b	0.37 ± 0.042 c
e22	Phenethyl acetate	103-45-7	3.88 ± 0.38 b	4.05 ± 0.11 b	17.07 ± 2.25 a	0.20 ± 0.025 c	3.34 ± 0.32 b
e23	Methyl caprate	110-42-9	nd	nd	nd	0.28 ± 0.077	nd
e24	Ethyl 3-hydroxyoctanoate	7367-90-0	nd	nd	1.86 ± 0.22 a	nd	0.42 ± 0.04 b
e25	Ethyl 9-decenoate	67233-91-4	nd	0.30 ± 0.036 a	nd	1.65 ± 1.06 a	nd
e26	Ethyl decanoate	110-38-3	2.11 ± 1.309 b	1.64 ± 0.33 b	0.73 ± 0.31 c	9.90 ± 2.28 a	3.30 ± 0.40 b
e27	Phenethyl butyrate	103-52-6	0.025 ± 0.013 b	0.24 ± 0.012 a	nd	nd	nd
e28	3-Methylbutyl nonanoate	7779-70-6	nd	nd	1.92 ± 1.25	nd	nd
e29	Ethyl laurate	106-33-2	0.51 ± 0.26 b	0.19 ± 0.10 b	0.27 ± 0.071 b	1.72 ± 0.79 a	0.15 ± 0.011 b
e30	Benzylcarbinyl caproate	6290-37-5	nd	0.60 ± 0.10 a	nd	nd	0.10 ± 0.039 b
Alcohols							
alc1	Pentanol	71-41-0	0.22 ± 0.095 a	nd	0.21 ± 0.19 a	nd	nd
alc2	2,3-Butanediol	513-85-9	0.098 ± 0.006 a	0.13 ± 0.047 a	nd	nd	0.096 ± 0.012 a
alc3	*(E)*-3-Hexen-1-ol	928-97-2	nd	0.098 ± 0.010 b	nd	0.24 ± 0.023 a	0.055 ± 0.006 c
alc4	Hexanol	111-27-3	14.61 ± 0.83 a	7.52 ± 0.15 c	13.49 ± 1.24 a	10.97 ± 1.61 b	7.37 ± 0.54 c
alc5	2-Ethylhexanol	104-76-7	0.25 ± 0.030 a	0.037 ± 0.005 c	0.13 ± 0.011 b	0.056 ± 0.014 c	nd
alc6	Benzyl alcohol	100-51-6	0.054 ± 0.011 c	0.21 ± 0.009 b	0.040 ± 0.007 c	1.27 ± 0.065 a	0.058 ± 0.007 c
alc7	1-Octanol	111-87-5	1.08 ± 0.053 a	0.81 ± 0.015 bc	0.57 ± 0.063 d	0.61 ± 0.16 cd	0.85 ± 0.12 b
alc8	Phenylethyl Alcohol	60-12-8	95.98 ± 7.74 a	22.87 ± 1.78 b	13.76 ± 1.10 c	13.00 ± 0.75 c	6.62 ± 0.62 c
alc9	Terpinen-4-ol	562-74-3	0.36 ± 0.013 a	0.28 ± 0.006 b	nd	nd	0.16 ± 0.028 c
alc10	*α*-Terpineol	98-55-5	nd	0.094 ± 0.005 b	0.19 ± 0.019 b	0.20 ± 0.054 b	2.95 ± 0.33 a
alc11	1,3-Octanediol	23433-05-8	1.06 ± 0.18 bc	1.55 ± 0.20 b	2.38 ± 0.43 a	nd	0.76 ± 0.045 c
alc12	Decanol	112-30-1	0.13 ± 0.012 b	0.15 ± 0.078 b	nd	0.31 ± 0.12 a	0.25 ± 0.043 ab
Aldehydes							
ald1	3-Furaldehyde	498-60-2	2.16 ± 0.021 a	1.75 ± 0.090 b	0.43 ± 0.018 d	1.95 ± 0.23 ab	0.67 ± 0.070 c
ald2	Benzaldehyde	100-52-7	0.45 ± 0.050 c	0.27 ± 0.016 d	0.14 ± 0.009 d	0.81 ± 0.057 b	1.13 ± 0.12 a
ald3	Phenylacetaldehyde	122-78-1	0.24 ± 0.052 a	0.06 ± 0.004 b	nd	nd	nd
ald4	Nonanal	124-19-6	0.35 ± 0.044 bc	0.48 ± 0.040 a	0.42 ± 0.055 ab	0.27 ± 0.053 c	0.34 ± 0.035 bc
ald5	Decanal	112-31-2	0.58 ± 0.15 a	0.59 ± 0.051 a	0.37 ± 0.027 a	0.47 ± 0.16 a	0.56 ± 0.13 a
Ketones							
k1	5-Hepten-2-one, 6-methyl-	110-93-0	nd	0.089 ± 0.003 b	nd	nd	0.12 ± 0.007 a
k2	Acetophenone	98-86-2	nd	nd	0.12 ± 0.013 a	0.032 ± 0.028 b	nd
k3	2-Nonanone	821-55-6	0.20 ± 0.011 b	0.24 ± 0.007 a	0.008 ± 0.007 d	nd	0.096 ± 0.014 c
k4	*β*-Damascenone	23726-93-4	0.067 ± 0.006 c	1.58 ± 0.092 a	0.10 ± 0.019 c	0.070 ± 0.030 c	0.57 ± 0.070 b
Acids							
acid1	2-Methylbutyric acid	116-53-0	0.18 ± 0.012 a	nd	0.076 ± 0.011 b	0.12 ± 0.008 b	0.26 ± 0.037 a
acid2	Hexanoic acid	142-62-1	2.46 ± 0.42 a	2.10 ± 0.091 a	nd	nd	1.11 ± 0.44 b
acid3	2-Ethylhexanoic acid	149-57-5	nd	nd	nd	nd	2.02 ± 0.22
acid4	Octanoic acid	124-07-2	7.81 ± 1.32 a	10.18 ± 1.27 a	nd	nd	nd
acid5	n-Decanoic acid	334-48-5	1.01 ± 0.36 b	9.48 ± 2.70 b	nd	nd	32.19 ± 8.81 a
acid6	Dodecanoic acid	143-07-7	nd	0.094 ± 0.012 b	0.24 ± 0.13 b	1.71 ± 0.93 a	0.17 ± 0.014 b
Others							
ot1	4-Ethylphenol	123-07-9	nd	1.66 ± 0.12 a	nd	nd	0.48 ± 0.036 b
ot2	4-Tert-Butylphenol	98-54-4	nd	nd	nd	1.01 ± 0.17	nd
ot3	Eugenol	97-53-0	0.049 ± 0.014 c	0.20 ± 0.021 b	0.61 ± 0.052 a	0.13 ± 0.098 bc	0.57 ± 0.014 a
ot4	Styrene	100-42-5	0.21 ± 0.037 b	0.39 ± 0.001 a	0.14 ± 0.004 c	0.14 ± 0.011 c	0.21 ± 0.020 b
ot5	D-Limonene	5989-27-5	nd	nd	0.024 ± 0.005 b	0.041 ± 0.026 b	0.22 ± 0.019 a
ot6	Myristicin	607-91-0	nd	nd	1.03 ± 0.14	nd	nd
Total			170.32	97.76	80.54	88.69	85.95

Values are the mean of three repetitions. nd: not detected. Different letters in the same row indicate significant differences at *p* < 0.05.

## Data Availability

The original contributions presented in the study are included in the article, further inquiries can be directed to the corresponding author.
